# Who Lives Longer, the Valve or the Patient? The Dilemma of TAVI Durability and How to Optimize Patient Outcomes

**DOI:** 10.3390/jcm13206123

**Published:** 2024-10-14

**Authors:** Vincenzo Cesario, Omar Oliva, Chiara De Biase, Alessandro Beneduce, Mauro Boiago, Nicolas Dumonteil, Didier Tchetche

**Affiliations:** 1 Groupe Cardiovasculaire Interventionnel, Clinique Pasteur, 45 Avenue de Lombez, CEDEX 3, 31076 Toulouse, France; vicesario91@gmail.com (V.C.); omaroliva93@gmail.com (O.O.); chiadebiase@gmail.com (C.D.B.); ale.beneduce@icloud.com (A.B.); mboiago@clinique-pasteur.com (M.B.); ndumonteil@clinique-pasteur.com (N.D.); 2 Cardiology Unit, Sant’Andrea Hospital, “Sapienza” University, Via di Grottarossa, 1035/1039, 00189 Rome, Italy

**Keywords:** aortic stenosis (AS), bioprosthetic valve failure (BVF), durability, structural valve degeneration (SVD), transcatheter aortic valve implantation (TAVI)

## Abstract

Over the past few years, transcatheter aortic valve implantation (TAVI) imposed itself as the first-choice therapy for symptomatic aortic stenosis (AS) in elderly patients at surgical risk. There have been continuous technological advancements in the latest iterations of TAVI devices and implantation techniques, which have bolstered their adoption. Moreover, the favorable outcomes coming out from clinical trials represent an indisputable point of strength for TAVI. As indications for transcatheter therapies now include a low surgical risk and younger individuals, new challenges are emerging. In this context, the matter of prosthesis durability is noteworthy. Initial evidence is beginning to emerge from the studies in the field, but they are still limited and compromised by multiple biases. Additionally, the physiopathological mechanisms behind the valve’s deterioration are nowadays somewhat clearer and classified. So, who outlasts who—the valve or the patient? This review aims to explore the available evidence surrounding this intriguing question, examining the various factors affecting prosthesis durability and discussing its potential implications for clinical management and current interventional practice.

## 1. Introduction

Surgical aortic valve replacement (SAVR) has represented the gold standard and the exclusive therapy for severe symptomatic aortic stenosis (AS) for a long time.

Over the last decade, transcatheter aortic valve implantation (TAVI) fired up the world of interventional cardiology, imposing itself as the first-choice therapy for AS in elderly patients.

Currently, this is true also in low-surgical-risk patients.

An analysis from the US Vizient Clinical Data Base, identifying 142,953 patients undergoing TAVI or SAVR for isolated aortic stenosis from 2015 to 2021 [1], showed that TAVI was adopted in 88% of cases in patients from 65 years old, and increased 2.7-fold in patients under 65 years old.

These data underscore the extremely high rise in TAVI treatments, and this is expected to increase.

Simultaneously to the widespread occurrence of patients undergoing transcatheter therapy for aortic valve disease, new challenges must be expected and faced in the near future. We should keep in mind that both surgical and transcatheter bioprostheses degenerate [2,3,4]. Concerning the “life-time valve journey”, valve durability is a key factor to consider.

Hereby, the focus of this paper is as follows: who lives longer, the valve or the patient? What are the future trends and challenges?

## 2. Cardiac Damage: A Not So Small Matter

In this era of expanding indications for TAVI, patient selection remains crucial. Device durability may become less relevant or even useless if the patient’s survival is shorter than the device’s lifespan. Currently, the indications for valve intervention are determined by the presence of severity parameters of valve disease in conjunction with patient symptoms. The importance of extra-valvular cardiac damage in the context of valvular intervention has been emphasized and linked to prognosis [5]. Cardiac damage has been proposed and categorized by echocardiography as follows: no extra-valvular cardiac damage (Stage 0), damage of left ventricle, systolic or diastolic disfunction or hypertrophy (Stage 1), left atrial or mitral valve damage (Stage 2), pulmonary hypertension or tricuspid valve damage (Stage 3), and right ventricular damage (Stage 4). Recent evidence suggests that a more accurate classification of cardiac damage, which integrates data from right heart catheterization and echocardiography, may enhance prognostic assessments [6]. Cardiac damage stratification has been demonstrated to be prognostically relevant irrespective of all hemodynamic subtypes of AS, with higher stages of damage linked to higher mortality rates. Moreover, cardiac damage stratification has been shown to be prognostically relevant across all hemodynamic subtypes of AS, with higher stages of damage associated with increased mortality rates [7]. Data indicate that if cardiac damage is identified at an early stage, as well as if valvular disease is detected early, appropriate treatment may lead to damage improvements and positively influence outcomes [8]. This latest study confirmed that the extent of damage at baseline is associated with the progression of damage and a dismal prognosis at the two-year follow-up, in contrast to patients with early-stage cardiac damage, who may also potentially show a reduction in damage, as previously mentioned. The early detection of valvular disease and the stage of cardiac damage are crucial for implementing the appropriateness of interventions for each patient. It should be emphasized that durability must be paired with optimal TAVI candidate selection, in order to minimize procedures that do not significantly impact patient prognosis.

## 3. Durability Definition

A shared and homogeneous definition of valve durability has been widely argued for by the scientific community to standardize studies’ outcomes. The pathway of a shared consensus was hampered by several obstacles. Firstly, a balanced definition between surgeons and interventional cardiologists was lacking. Due to the inconsistency of the definition, for many years, the need for redo surgeries has been the pragmatic endpoint for considering the failure of bioprostheses in surgical procedures [9]. Intuitively, this definition underestimates the effective incidence of prosthesis failure, since many patients were not always eligible for a new surgical intervention.

On the other hand, the caveat for transcatheter therapies is that the “first generation” of patients treated by TAVI, namely a group of high-risk octogenarians, died before the good functioning of the bioprosthesis could be addressed at a long follow-up.

For the first time, in 2017, a shared definition of structural valve deterioration (SVD) had been proposed and adopted by the European Association of Percutaneous Cardiovascular Interventions (EAPCI) and endorsed by the European Society of Cardiology (ESC) and by the European Association for Cardio-Thoracic Surgery (EACTS) [10].

**Figure 1:**
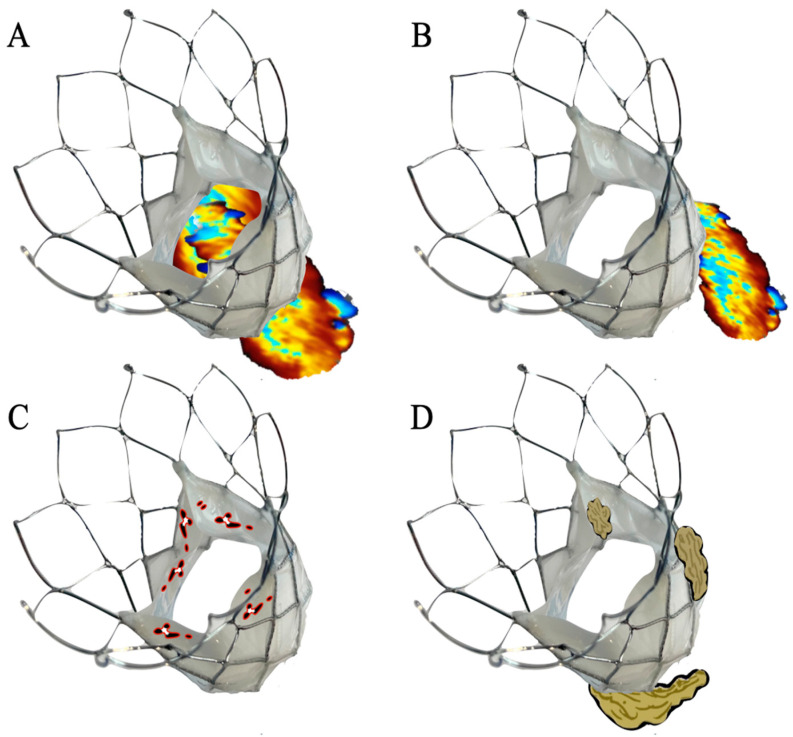
The four categories of bioprosthetic valve dysfunction. (**A**): Structural valve deterioration is a deterioration process that directly involves the valvular components leading to prosthesis stenosis or regurgitation (e.g., fibrotic degeneration, leaflet tear). (**B**): Non-structural valve dysfunction (NSVD) (e.g., para-prosthetic regurgitation). (**C**): Thrombosis. (**D**): Endocarditis. Parallel to the BVD pathological categories, three stages of dysfunction, based mainly on the mean gradient (MG), the effective orifice area (EOA), the Doppler velocity index (DVI), and intraprosthetic aortic regurgitation (AR), have been proposed (table one).

In 2018, another definition on valve degeneration was proposed based on Valve-in-Valve International Data (VIV-ID) [11]. The VIVID definition suggested three levels of SVD severity based on morphological and hemodynamic features. Stage three represents the prosthesis’ failure with an indication for reintervention. More recently, Generaux et al. reported an update on SVD based on the Valve Academic Research Consortium 3 (VARC-3) criteria [12]. In this last document, the authors outlined the central role of echocardiographic diagnoses of prosthesis failure. Given the hemodynamic state fluctuations, the need for almost two consecutive echocardiograms is highlighted in order to give a definite diagnosis. Four categories of bioprosthetic valve dysfunction (BVD) are proposed (Figure 1):

- Structural valve deterioration: this may be conceived as a deterioration process that directly involves the valvular components as a calcific or fibrotic degeneration or a traumatic process (e.g., leaflet tear or stent fracture).
- Non-structural valve dysfunction (NSVD): any process that is not directly linked to the prosthesis itself; the clearest example for this category is para-prosthetic regurgitation.
- Thrombosis or hypo-attenuated leaflet thickening (HALT).
- Endocarditis.

Parallel to the BVD pathological categories, three stages of dysfunction, based mainly on the mean gradient (MG), the effective orifice area (EOA), the Doppler velocity index (DVI), and intraprosthetic aortic regurgitation (AR), have been proposed (Table 1).

Moreover, a classification expressing the clinical status of BVD was declined in two categories, subclinical and bioprosthesis valve failure (BVF). The former, which represents the unfavorable final consequence of BVD, has three stages (Table 2):

The introduction of standardized definitions is widely accepted and adopted in clinical trials. This aspect has played a pivotal role in shaping the data about prosthesis performance, spanning up to an eight-year follow-up.

## 4. Durability Causes

Several factors determining valve durability can be theorized, even if some of them are not well understood. The heart valves are characterized by a histological architecture in which three components of the valve tissue (fibrosa, spongiosa, and ventricularis) act as an active network to provide elasticity and resistance to hemodynamic stress [13].

Certainly, prostheses cannot rely on this efficient structure; indeed, the leaflet materials are typically xenografted (made from porcine or swine tissue), and for this reason, they tend to degenerate sooner than native valves [14].

**Figure 2:**
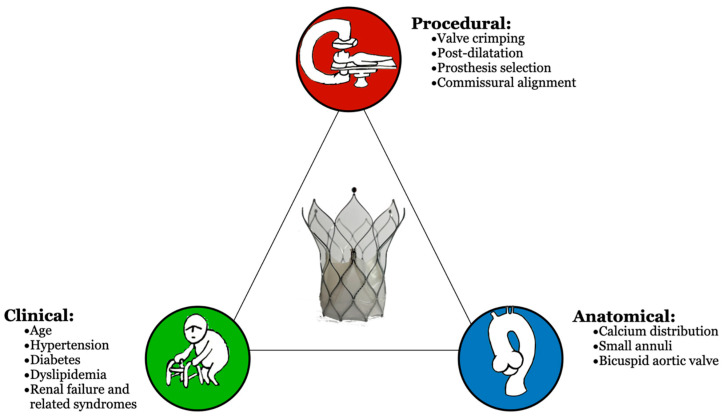
The triangle of factors influencing valve durability.

Three categories of elements that influence prosthesis deterioration are illustrated in Figure 2: clinical, anatomical, and procedure-related deterioration.

Concerning the clinical factors, the most prevalent influence on degeneration is being at a young age at the time of implantation [15]. This holds true for both transcatheter and surgical valves.

Clinical host-mediated factors that can contribute to the prosthesis deterioration process are the common “risk factors”, including atherosclerosis, hypertension, diabetes mellitus, and renal failure, with all the consequent syndromes such as hyperparathyroidism or hypercalcemia [16,17].

The dystrophic calcification process is the pathological process through which calcium phosphates are deposited on the prosthesis, which helps in determining their aging and dysfunction status.

This process has been described as a passive mechanism but also as an active, immune-mediated process [18]. Further studies have addressed the role of immune responses in prosthesis degeneration. The presence of immune cells, predominantly macrophages, and their products highlight the possible inconsistency of glutaraldehyde treatments in preserving valves from the immune response [19,20]. From an anatomical perspective, a bulky calcium distribution could play a central role on the functioning of the prosthesis, with a negative impact on heart valve durability [21]. THVs are placed within an anatomical position marked by calcification and fibrosis, which may exhibit different degrees of asymmetry and distribution. In general, calcium can be present at the level of the annulus, within the cusps, distributed along the commissure, within the left ventricular outflow tract, and in the aortic root-free wall. The distortion of the prosthesis, derived from the inappropriate valve overture due to calcium, could generate flow turbulence and mechanical stress. These improper hemodynamic conditions make valve tissues more susceptible to calcification, resulting in tissue degeneration and prosthesis dysfunction [22].

Another crucial factor influencing prosthesis hemodynamics is the dimensions of the baseline annulus and the prosthesis size, with a potential mismatch between the two sizes. Patient prosthesis mismatch (PPM) can occur when an inappropriate small valve for the native annulus, with a smaller indexed orifice area (EOA), is implanted within the aortic annulus and is not fit for the patient’s body surface area (BSA) [23]. The immediate hemodynamic condition results in increased trans-prosthetic gradients and in accelerated valve degeneration [24]. The SMALL-TAVI 2 study, an international, multicenter, retrospective registry, addressed this topic [25]. The aim of the study was to compare the hemodynamic performance of contemporary transcatheter prostheses in patients with severe aortic stenosis and a small annulus (perimeter < 72 mm or area < 400 mm^2^). The pre-discharge mean gradient and incidence of PPM were the selected hemodynamic parameters to evaluate. Data from 1378 patients were collected. Both supra-annular (SA) and intra-annular (IA) self-expanding valves (SEVs) and balloon-expanding valves (BEVs) have been analyzed. TAVI with a SA SEV yielded lower mean aortic gradients and a lower incidence of PPM. These results endorse the implantation of SA SEVs in patients with small annuli.

In this setting, the SMall Annuli Randomized To Evolut or SAPIEN (SMART) Trial was the first randomized study comparing two of the most utilized transcatheter aortic valve prostheses (i.e., Evolut and Sapien platform) in subjects with this peculiar anatomy [26]. The investigators randomized 716 patients with symptomatic severe AS and a small aortic valve annulus area (430 mm^2^ or less) to one of the two groups.

At 12 months, the Kaplan–Meier estimate for the primary endpoint (a composite of death, disabling stroke, or hospitalization for heart failure) was 9.4% for the SE valve and 10.6% for the BE valve (*p* < 0.001 for noninferiority). During the same time frame, the Kaplan–Meier estimate for BVD was 9.4% for the SE valve and 41.6% for the BE valve (*p* < 0.001 for superiority). This finding might appear surprising, but it is expected given that at 30 days, moderate or severe PPM was found in 11.2% of patients with the SE valve and in 35.3% of those with the BE valve (*p* < 0.001).

The study showed that in this population, in which the majority were women, a supra-annular SE valve offers superior hemodynamic performance compared to the BE intra-annular prosthesis with no differences in the clinical endpoint at the 12-month follow-up.

While no differences in clinical outcomes were observed at the 1-year follow-up, long-term follow-ups are ongoing to assess whether these early hemodynamic advantages will lead to clinically significant outcomes.

In addition to the native annulus dimension, the aortic valve native anatomy could play a role in terms of long-term device durability. This could be the case for a bicuspid aortic valve (BAV) anatomy due to its unique features. BAVs are characterized by two anatomical or functional cusps, by a different number of commissures, by the presence of diffuse and asymmetric calcifications (commonly involving left coronary cusp), elongated leaflets and, possibly, by the presence of a raphe [27,28]. These anatomical features predispose the transcatheter valves to under-expansion, asymmetry, and a higher post-procedural gradient [29]. The Bicuspid Aortic Valve Anatomy and Relationship With Devices (BAVARD) registry is, at this time, one of the largest registries addressing sizing ratios and valve morphology in patients with a bicuspid anatomy undergoing TAVI with SE valves [30]. One of the main findings of the study was the constant prostheses under-expansion in patients with a BAV anatomy at the post-procedural CT analysis. The average prosthesis diameters were consistently smaller than the mean diameter of the aortic annulus at baseline. Echocardiographic data at 30 days showed a significantly smaller indexed orifice area in patients with BAV compared to patients with a tricuspid anatomy, even if no differences were reported in terms of PPM. The authors underscored the importance of an appropriate CT analysis in BAV patients, which is another issue that a bicuspid anatomy poses, but the longer follow-up is conducted at one year. As a matter of fact, in the BIVOLUTX study, supra-annular SEVs were systematically adopted in patients with BAVs, demonstrating favorable hemodynamic valve performance [31]. The study indicated that the proper selection of the prosthesis is crucial to ensure optimal hemodynamic performance and preserve long-term durability, but the available data are from the one-year follow-ups.

Finally, there are procedural factors that can influence the final expansion of the prostheses and their longevity. The compressive stress that all the valve components undergo during the crimping phase, and during the recapture for the SEV, could determine traumatic leaflet and stent damage [32]. The prosthesis stress during the crimping phase could depend on factors such as the leaflet thickness, material properties, and the ratio of the leaflet volume to the available volume inside the crimped valve [33]. In recent years, modified specific delivery system insertion and rotation techniques have been presented in order to obtain commissural alignment. The transcatheter heart valve alignment has the potential to impact the prosthesis orientation in order to ensure future coronary access [34].

Commissural alignment could hypothetically also influence valve durability [35].

In misaligned valves, alterations in fluid dynamics have been evoked and theorized.

Non-physiological vorticity can lead to heightened blood stagnation within the sinus of the Valsalva, determining thrombus formation and promoting prosthesis degeneration [36,37].

In the RESOLVE (Assessment of TRanscathetER and Surgical Aortic BiOprosthetic Valve Thrombosis and Its TrEatment With Anticoagulation) study, commissural alignment, evaluated with a post-procedural CT, was shown to influence both the device’s performance and the clinical outcomes in patients with severe aortic stenosis treated by TAVI with BE valves [38].

Post-dilatation (PD) is a maneuver adopted after valve deployment in order to optimize prosthesis implantation, especially concerning paravalvular regurgitation (PVR) and moderate or severe PPM [39,40]. Concerns about the safety of PD are raised in relation to potential prosthesis damage that could thus impact durability [41].

J.S. Sanchez et al. analyzed data from the Clinical Service data repository (a nation-based data repository and medical care project) on patients undergoing SE TAVI with the aim of assessing the influence of PD on valve durability [42].

The study showed that at 6-years follow-up (FU) no differences were observed in terms of clinical outcomes or SVD between patients who underwent PD and those who did not.

The knowledge of these variables highlights the importance of a careful selection process to choose the most suitable valve for each patient in order to mitigate the impact of the different factors that could adversely affect the durability of the prosthesis.

### 4.1. Evidence from 1 to 5 Years

As mentioned above, limited data are available on TAVI durability, particularly for first-generation devices. The primary explanation is that participants in the trial assessing the first-generation prostheses were identified as high-risk candidates for surgery, with a notable decrease in their life expectancy.

The PARTNER trials explored the comparisons between the Sapien BE-TAVI and SAVR across various categories of surgical risk.

The PARTNER 1A trial was the first study enrolling high-surgical-risk patients randomized to TAVI with BE valves or SAVR [43]. At the 5-year FU, no cases of SVD necessitating SAVR were observed in either group. Nevertheless, moderate/severe aortic regurgitation occurred more frequently in the TAVI group (14%) than in the SAVR group (1%), and it was associated with an increased risk of mortality in the former group.

The PARTNER 2A trial enrolled intermediate-risk patients, randomizing them in the same fashion of the previous study adopting the second-generation SAPIEN XT BE valve [44]. Freedom from SVD was higher in the SAVR group compared to the Sapien XT group at the 5-year FU (90.5% vs. 96.5%) [45]. Moreover, the rate of mild paravalvular aortic regurgitation was higher in the TAVI group (33.3% vs. 6.3%).

Simultaneously, the CoreValve U.S. Trial has compared TAVI vs. surgery in a high-risk cohort of patients with aortic stenosis with a SE valve, showing the superiority of TAVI in terms of all-cause mortality [46,47].

The 5-year FU demonstrates no difference between surgery and TAVI in terms of primary endpoint; moreover, the occurrences of severe SVD and valve reinterventions were infrequent [48].

In this specific context, the echocardiographic analysis showed a constant superiority of TAVI over SAVR in terms of EOA and mean gradients (7.1 ± 3.6 mmHg for TAVI and 10.9 ± 5.7 mm Hg for SAVR). The rate of AR was higher in patients treated with TAVI.

The overall incidence of SVD was infrequent and comparable across the treatment groups, with the surgical group experiencing a higher rate of moderate SVD (26.6% vs. 9.2%; *p* < 0.001).

Comparable results in terms of hemodynamic performance at two and five years have been observed in the SURTAVI trial, in which intermediate-risk patients were randomized to SE TAVI or SAVR [49,50].

Patients with severe aortic stenosis at a low surgical risk undergoing SAVR or TAVI with BE valves were enrolled in the PARTNER 3 trial [51]. Patients who underwent TAVR exhibited a notably reduced incidence of the primary endpoint (a composite of death, stroke, or rehospitalization) at 1 year.

At five years, 86.3% of patients treated with TAVI and 87.4% of patients treated with surgery were alive with normal prosthesis parameters [52].

The hemodynamic evaluation, based on VARC 3 criteria, showed a rate of BVF of 1.4% in the TAVR group and of 2.0% in the SAVR group, with reintervention needs occurring in 2.2% of patients for the former and 2.6% for the latter. It is advisable to emphasize that the rate of AR and PVR resulted to be anew higher in patients who underwent TAVI.

The Evolut Low Risk trial enrolled patients who were at a low surgical risk, with aortic stenosis and indication to valve replacement, randomizing them to either TAVI with the SE valve or SAVR [53].

All-cause mortality and disabling stroke, the two elements of the primary endpoint, was lower in patients treated with TAVI. These results remained consistent during the extended FU, which was documented in the recently published three-year follow-up analysis [54].

At three years, there was no difference in terms of reintervention (1% in patients treated with TAVI vs. 0.9% of surgical patients). What is remarkable is the excellent hemodynamic performance of SE valves. The EOA was greater in transcatheter prostheses than in surgical valves (2.2 cm^2^ vs. 2.0 cm^2^; 95% CI: 0.2–0.3; *p* < 0.001) and it was accompanied by lower mean gradients (9.1 mmHg vs. 12.1 mmHg; 95% CI:3.6 to 2.4; *p* < 0.001). A moderate PPM has turned out to be superior in patients who underwent surgery (25.1% vs. 10.6%).

This evidence holds utmost significance when considering the potential role that PPM might have in determining SVD and its progression to BVF.

A recent study comparing TAVI and surgery in 4762 patients (2099 randomized patients and 2663 nonrandomized) showed that the rate of SVD is higher in surgical patients (4.38 vs. 2.20), and this is strongly linked to all-cause mortality and hospitalization for valve disease or worsening heart failure, corroborating the data that have already emerged from the described studies [55].

Some evidence comparing SE and BE prostheses are available.

Interesting results came up from the 5-year FU of the CHOICE trial [56].

This was a study in which 241 patients at a high surgical risk with severe symptomatic aortic stenosis were randomized to TAVI with BE or SE valves.

There were no differences observed between the two treatment groups in terms of overall mortality, cardiovascular-related mortality, strokes, and recurring hospitalization for heart failure.

Notably, the hemodynamic performance of SE valves was better with a higher valve area (1.6 ± 0.5 cm^2^ vs. 1.9 ± 0.5 cm^2^; *p* = 0.02) and consequently with lower mean trans-prosthetic gradients.

However, the favorable hemodynamic profile did not translate into a clinical advantage. Although moderate or severe SVD happened more frequently with the BE valves, the rate of BVF was minimal, with no significant difference observed between the two groups (4.1% vs. 3.4%; *p* = 0.63).

Interesting conclusions have emerged from the results of the Nordic Aortic Valve Intervention (NOTION)-2 trial, published this year [57]. This study randomized low-risk patients aged 75 years or younger with severe AS to either TAVI or surgery. Notably, for the first time, patients with a bicuspid anatomy were included in a randomized trial, making the findings particularly compelling.

At the 1-year follow-up, no differences were observed in the primary endpoint (all-cause death, stroke, or rehospitalization) between the two groups in the entire cohort.

In the bicuspid group, the incidence of the same endpoint was lower in those who underwent surgery compared to TAVI, primarily due to the higher rate of moderate or greater PVR in the TAVI population. While these findings reaffirm the strong performance of TAVI in younger, low-risk patients, they also raise a cautionary note regarding bicuspid anatomies. The concern is that current technical or strategic limitations could compromise long-term durability, which is a critical factor, especially in younger patients.

### 4.2. Evidence from 5 to 10 Years

Patient selection, pre-procedural work-up, device availability, and implantation techniques have all undergone substantial changes in recent years. No conclusive or reliable randomized data on valve durability exist beyond the 10-year time frame. The Nordic Aortic Valve Intervention (NOTION) trial randomized patients with severe AS at a lower surgical risk to TAVI with the SE prosthesis or SAVR [58]. There was no difference for major outcomes (rate of all-cause mortality, stroke, or myocardial infarction) at 5 years. At the 8-year FU of the trial, there were no differences in terms of primary outcomes [59]. Additionally, the SVD risk was significantly reduced in patients treated with TAVI compared to SAVR (13.9% vs. 28.3%; *p* = 0.0017), while BVF incidence showed no significant difference (8.7% vs. 10.5%; *p* = 0.61). Recently, the NOTION trial reached the 10-year milestone [60]. Concerning primary outcomes, no differences were reported. Here too, the occurrence of SVD was notably lower in patients who underwent TAVI as opposed to those who received SAVR. Severe SVD was observed in 1.5% and 10.0% of cases (HR 0.2; 95% CI 0.04–0.7; *p* = 0.02) following TAVI and SAVR, respectively. The cumulative occurrence of severe NSVD was 20.5% and 43.0% (*p* < 0.001), while for endocarditis, no significant differences occurred. There were no cases of clinical valve thrombosis. BVF was described in 9.7% and 13.8% after TAVI and SAVR, respectively (HR 0.7; 95% CI 0.4–1.5; *p* = 0.4). Interestingly, over 98% of TAVI patients were implanted with a THV ranging from 26 to 31 mm, whereas 98% of SAVR patients received a bioprosthesis with sizes from 19 to 25 mm. As a result, a greater number of SAVR patients experienced PPM and elevated trans-prosthetic gradients, which account for the elevated rates of NSVD.

While the results of this trial are reassuring, they do not provide definitive conclusions about the long-term durability of TAVI.

Several limitations must be considered. Firstly, only 25% of patients remained alive at 10 years, which will be a significant concern for all the studies involving first-generation prostheses. Additionally, the TAVI arm of the study used only first-generation SEVs, while the SAVR arm included 35% of valves that were either Trifecta (Abbott) or Mitroflow (Sorin) valves, both of which have known durability issues [61,62].

The CoreValve first-generation SE prosthesis durability at the 8-year mark has been analyzed on 990 inoperable or high-risk patients. Data were extrapolated and analyzed from the Clinical Service Project database [63]. No differences were reported in terms of trans-prosthetic gradient at baseline and at the 8-year echocardiographic FU. The rate of moderate and severe SVD were 3.0% and 1.6%, respectively. BVF was recognized in 2.5% of patients. These data came from one of the longest available FUs with the first-generation CoreValve SE bioprostheses. Considering the absence of an echocardiographic core lab as one of the potential sources of bias, a more optimistic perspective should be adopted, taking into consideration enhanced operator experience and advancements in prosthetic technology that occurred in recent years. The most significant studies and trials referenced above are listed in Table 3.

## 5. Treatment for Transcatheter Aortic Valve Failure

**Figure 3:**
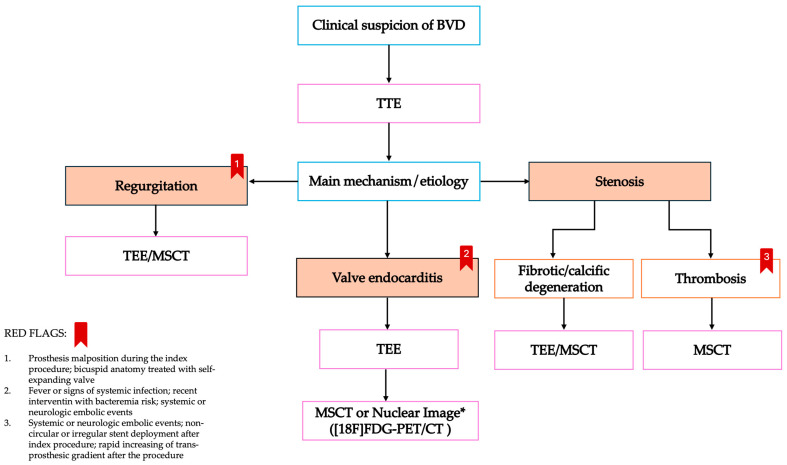
Diagnosis flowchart in case of suspicion of bioprosthesis valve dysfunction. BVD: bioprosthesis valve dysfunction; MSCT: multi-slice computed tomography; TEE: trans-esophageal echocardiography; TTE: trans-thoracic echocardiography; [18F]-FDG-PET: [18]-fluorodeoxyglucose-positron emission tomography. *: nuclear imaging may be applicable in case of high clinical suspicion of endocarditis with inconclusive TTE/TEE.

Treatment decisions, including the consideration of a redo intervention, should be made after a proper clinical assessment and discussed with the local heart team (Figure 3). Overall, reported reintervention rates are low, with less than 15% at the 20-year mark, and with variations based on several factors including the patient’s age as well as the model of the selected prosthesis [64,65,66].

A recent meta-analysis involving 7970 patients from seven trials with follow-up periods ranging from 2 to 8 years compared the durability of TAVI and SAVR [67]. No significant difference was found between the two groups regarding SVD. However, the TAVI cohort showed a statistically significant higher risk of reintervention (OR 2.03; 95% CI: 1.34–3.05) and moderate-to-severe AR (OR 6.54; 95% CI: 3.92–10.91) compared to the SAVR group. As a matter of fact, the TAVI cohort demonstrated a better hemodynamic profile, with lower trans-prosthesis gradients and a larger effective orifice area.

Basically, in addition to a BVF with a clinical need for reintervention, there are two procedures available: perform a new TAVI, referred as a redo TAVI or TAVI-in-TAVI, the or surgical removal of the failed valve, followed by the implantation of a new prosthesis.

Certainly, the choice between these two options depends on several factors, with the primary consideration being the nature of the valve failure, like the under-expansion of the valve, which may impact the hemodynamics of the second valve [68].

Failures due to conditions like endocarditis typically leave few options, with surgery often being the only viable choice.

The type of the initially implanted valve and its implantation strategy (such as commissural alignment and the implantation depth) will significantly influence the choice of intervention and the type of valve due to concerns about coronary obstruction, coronary re-access, PPM, and the opportunity to perform techniques such as Bioprosthetic or Native Aortic Scallop Intentional Laceration to Prevent Iatrogenic Coronary Artery Obstruction (BASILICA) [69,70,71,72,73].

Surgical interventions following TAVI are associated with high rates of mortality and morbidity due to various elements [74]. Among these, the neo-endothelization of the valve within the aortic root presents a significant challenge, requiring aortic repair or replacement in many cases [75].

Interesting data emerged from the international EXPLANTORREDO-TAVR registry, which compared TAVI surgical interventions to TAVI-in-TAVI interventions [76].

The analysis revealed that TAVI explants had a shorter median time to reintervention, with PPM being the first cause of failure and higher mortality rates at 30 days, 13.6% vs. 3.4% (*p* < 0.001), and at 1 year, 32.4% vs. 15.4% (*p* ≤ 0.001).

In the EXPLANT-TAVR registry, which compared outcomes in patients undergoing transcatheter prosthesis surgery between failed SE and BE valves, the primary reason for reintervention was endocarditis, and no significant differences in mortality were observed between the two types of prostheses at three years [77,78].

The mortality rate for surgical explants was high, with 16% at 30 days and 33% at 1 year.

While surgery is associated with this high mortality rate, the same cannot be said for the TAVI-in-TAVI procedure.

Therefore, despite the limited data, TAVI-in-TAVI has shown good short-term outcomes and safety, with a high survival rate at one year [79].

The primary concern with the TAVI-in-TAVI procedure is the risk of a compromised coronary flow and future coronary access. The risk arises from the formation of a neoskirt, which is created when the leaflets of the first implanted valve are pinned open by the second implanted prosthesis [80,81].

Another mechanism evoked as a risk for coronary obstruction is the sinus of the Valsalva sequestration, which occurs when the neoskirt extends above the sinotubular junction or when the distance between the valve frame and the STJ is less than 2 mm [82].

The redo TAVI risk may be mitigated by a careful CT analysis, which serves as the best tool to apply the best strategy in order to avoid the risks associated with the procedure and also to ensure the optimal prosthesis selection [83].

Another cause of BVF that may require different treatment from surgery or TAVI in TAVI is valvular thrombosis.

This may manifest as two distinct entities: subclinical leaflet thrombosis (SLT), commonly diagnosed as hypo-attenuated leaflet thickening (HALT), and clinical valve thrombosis (CVT), which is a rare entity [84].

There are no definitive recommendations for treating this condition due to the paucity of data; however, oral anticoagulation treatments and thrombolytics are considered the first-line treatments. Recently, a treatment algorithm has also been proposed [85,86].

With the rising prevalence of valvular heart disease and the growing potential for recognizing conditions like prosthetic deterioration, the role of heart valve clinics is becoming increasingly crucial [87]. The complex clinical scenarios involving SVD or BVF, along with the need for reintervention, require a high standard of care that a well-organized clinic can provide. The early recognition of each symptom and the prompt detection of prosthetic dysfunction play a significant prognostic role, as recent evidence has shown [88].

Over the past year, growing evidence has highlighted the negative impact of moderate aortic stenosis on clinical outcomes, and several clinical trials are currently underway [89,90]. In this context, heart valve clinics have shown a positive impact on outcomes through early symptom detection and timely recommendations for valve interventions [91,92].

## 6. Conclusions

We are witnessing a philosophical shift in the treatment of aortic stenosis. Treatment options are now expanding beyond boundaries that were once considered prohibitive. Today, TAVI is a globally adopted therapy, applicable across all age groups, surgical risks, and anatomical variations. The longevity of transcatheter heart valves is a matter of concern when considering the extension of TAVI adoption.

According to the latest trials on younger and low-surgical-risk patients, the application of TAVI extends over high and intermediate-surgical-risk patients. TAVI results, at the very least, are comparable to SAVR in treating low-risk surgical candidates.

Nevertheless, the results of low-risk trials are not universally applicable to all young, low-risk individuals. In these cases, AS treatment should be viewed as a lifelong management strategy, taking into account factors such as the potential need for new valve interventions (e.g., TAVI in TAVI) and personal patient preferences [93].

For these reasons, the durability of the bioprosthesis is of paramount importance.

Nowadays, the evidence is scarce. Data derived from studies involve high-risk patients and first-generation devices implanted by operators in the process of learning and gaining experience.

The next RCT to achieve a 10-year follow-up is the PARTNER 2A trial, which involves an elderly population with a high or intermediate surgical risk, and which will be biased by a significant attrition rate at 10 years.

The extended outcomes from the Evolut low risk and Partner 3 trials will establish a foundation of evidence for the field, providing a more current and robust understanding of the TAVI patient population, and the limits and strengths of this treatment.

## Figures and Tables

**Table 1:** Stages of prosthesis dysfunction.

Stage	MG (mmHg)	EOA (cm^2^) Decrease	DVI	Intraprosthetic AR
1	No significant changes	No significant changes	No significant changes	No significant changes
2	≥10, resulting in MG ≥20	≥0.3 or ≥25%	≥0.1 or ≥20%	New occurrence, or increase of 1 grade, resulting in ≥ moderate AR
3	≥20, resulting in MG ≥30	≥0.6 or ≥50%	≥0.2 or ≥40%	New occurrence, or increase of 2 grades, resulting in ≥ severe AR

AR, aortic regurgitation; DVI, Doppler velocity index; EOA, effective orifice area; MG, mean gradient.

**Table 2:** Bioprosthesis valve failure stages.

Stage	Features
1	Any clinical manifestation of BVD or hemodynamic valve deterioration stage 3
2	Aortic valve reintervention (surgical or percutaneous)
3	Valve-related death

BVD: bioprosthesis valve dysfunction.

**Table 3:** Summary characteristics of TAVI studies and trials concerning valve durability.

Study	Study Design	Patients, n TAVI vs. SAVR	Age TAVI vs. SAVR	STS TAVI vs. SAVR	Prosthes	Follow-Up, Years	Results/Interpretation
PARTNER 1A	Randomized trial	348 vs. 351	83.6 ± 6.8; 84.5 ± 6.4	11.8 ± 3.3; 11.7 ± 3.5	BE vs. SAVR	5	No cases of SVD. Rate of moderate/severe AR was higher in TAVI cohort
PARTNER 2A	Randomized trial	1011 vs. 1021	81.5 ± 6.7; 81.7 ± 6.7	5.8 ± 2.1; 5.8 ± 1.9	BE vs. SAVR	5	Freedom from SVD resulted higher in the SAVR group. Rate of mild PVR was higher in TAVI cohort
CoreValve U.S.	Randomized trial	390 vs. 357	83.2 ± 7.1; 83.5 ± 6.3	7.3 ± 3; 7.5 ± 3.2	SE vs. SAVR	5	Higher rate of moderate SVD in surgery cohort. Rate of moderate/severe AR was higher in TAVI cohort
SURTAVI	Randomized trial	864 vs. 796	79.9 ± 6.2; 79.7 ± 6.1	4.4 ± 1.5; 4.5 ± 1.6	SE vs. SAVR	5	No difference between the groups in terms of SVD
PARTNER 3	Randomized trial	503 vs. 497	73.3 ± 5.8; 73.6 ± 6.1	1.9 ± 0.7; 1.9 ± 0.6	BE vs. SAVR	5	BVF of 1.4% in the TAVR group and of 2.0% in the SAVR group
Evolut Low Risk	Randomized trial	734 vs. 734	74.1 ± 5.8; 73.6 ± 5.9	1.9 ± 0.7; 1.9 ± 0.7	SE vs. SAVR	3	No difference in terms of reintervention. Rate of PPM superior in surgery cohort
CHOICE	Randomized trial	121 vs 120 (BE vs. SE)	81.9 ± 6.7; 79.6 ± 15.8 (BE vs. SE)	5.6 ± 2.9 6.2 ± 3.9 (BE vs. SE)	SE vs. BE	5	Moderate or severe SVD occurred more frequently with BE prosthesis
NOTION-2 *	Randomized trial	187 vs. 183	71.1 ± 3.1; 71.0 ± 3.2	1.1 (0.9 − 1.5) 1.1 (0.8 − 1.5)	SE vs. SAVR	3	No difference between the groups in terms of SVD
NOTION	Randomized trial	145 vs. 135	79.2 ± 4.9 79.0 ± 4.7	2.9 ± 1.6 3.1 ± 1.7	SE vs. SAVR	10	SVD was lower in patients who underwent TAVI
Testa et al. [63]	Registry	990	82 ± 6	9 ± 10	SE	8	Low cumulative incidence of BVF and SVD

BE: balloon-expanding; BVF: bioprosthesis valve failure; PVR: perivalvular regurgitation; SAVR: surgical aortic valve replacement; SE: self-expanding; STS: Society of Thoracic Surgeons Score; SVD: structural valve deterioration; TAVI: transcatheter aortic valve implantation. *: It was the first trial that randomized patients with a bicuspid anatomy.

## Abbreviations

AS	aortic stenosis
BAV	bicuspid aortic valve
BE	balloon expandable
BVD	bioprosthetic valve dysfunction
BVF	bioprosthetic valve failure
CT	Computed tomography
DVI	Doppler velocity index
EOA	effective orifice area
LVOT	left ventricular outflow
MAEs	major adverse events
MSCT	multi-slice computed tomography
PD	Post-dilatation
PM	pacemaker
PPM	patient-prosthesis mismatch
PVR	perivalvular regurgitation
SAVR	surgical aortic valve replacement
SVD	structural valve deterioration
THVs	transcatheter heart valves
TAVI	transcatheter aortic valve implantation
VARC-3	Academic Research Consortium 3
